# Correction: Deficient pulmonary IFN-β expression in COPD patients

**DOI:** 10.1371/journal.pone.0219349

**Published:** 2019-07-01

**Authors:** José García-Valero, Jordi Olloquequi, Juan F. Montes, Esther Rodríguez, Mireia Martín-Satué, Laura Texidó, Jaume Ferrer Sancho

## Notice of Republication

This article was republished on June 21, 2019, to correct errors in the display of the Greek symbol β throughout the article. Please download this article again to view the correct version. The originally published, uncorrected article and the republished, corrected articles are provided here for reference.

## Supporting information

S1 File Originally published, uncorrected article. (PDF)

S2 File Republished, corrected article. (PDF)
