# Supplementary material for: Deficient pulmonary IFN‐β expression in COPD patients
Source: PLoS One. 2019 Jun 6;14(6):e0217803. doi: 10.1371/journal.pone.0217803 (PMC6553750; doi:10.1371/journal.pone.0217803)
Supplement: S1 Text — (DOCX) [file pone.0217803.s001.docx]

**Supporting Information S1**

**Supplement for Material and Methods section**

**Subjects**

The study was approved by the ethics committee of the Vall d'Hebron University Hospital, Barcelona, Spain (certificate of ethical approval PI 040635), and all subjects provided written informed consent. Seventy subjects, recruited between 2005 and 2010, who underwent lung resection for non-obstructive peripheral lung tumors or were subjected to lung transplantation for very severe COPD were studied (Table 1). Non-COPD groups were recruited consecutively among patients undergoing lung surgery for lung cancer. Patients with other respiratory conditions than lung cancer were excluded. Standard procedures and equipment (Masterlab; Jaeger, Würzburg, Germany) were used to assess pulmonary function in all patients, including measurements of forced expiratory volume in 1 s (FEV1), forced vital capacity (FVC), residual volume (RV) and total lung capacity (TLC). All subjects had been free of acute lung infections and none had received chemotherapy before surgery. COPD diagnosis was based on spirometry according to ERS recommendations and non-COPD patients were divided into smokers (current or ex-smokers), and non-smokers. We evaluated anti-viral IFN- response in lung samples from 70 subjects (9 control never smokers, 19 control smokers without COPD, 21 patients with moderate COPD and 21 patients with very severe COPD) by immunohistochemical techniques and quantitative real-time PCR.

Table 1 shows the number of subjects, demographic and smoking characteristics, spirometry variables and macroscopic emphysema degree in each group.

**Tissue sample processing for Light Microscopy**

The resected lungs or lobes obtained in surgery were immediately inflated at 30 cm of water pressure for 1 hour with 4% (w/v) formaldehyde in 0.1 M phosphate-buffered saline solution (pH 7.4), prior to immersion in fixative for 24 hours. After fixation, those surgical specimens destined to be analyzed by light microscopy techniques were sliced serially into 1-cm axial sections, then the macroscopic severity of emphysema was graded using the panel grid described by Thurlbeck and coworkers (1970). After that, 2 x 2 x 1 cm blocks were uniformly random sampled (Nyengaard, 2006; Hsia, 2010), embedded in paraffin, cut into serial 4-µm sections and mounted on positive charged Starfrost Plus slides (Menzel-Gläser, Braunschweig, Germany). One section per block was stained with hematoxilyn and eosin. Sections were examined with a fully automated Olympus BX61 microscope and images recorded with a DP70 digital camera.

**Immunohistochemistry**

Deparaffinized sections were processed at 121°C in a 2100 Retriever (PickCell Laboratories, Leiden, Netherland) in Tris-HCl 10 mM-EDTA 0.5 mM pH 9.0 buffer (IFN-β, MDA-5), or in EDTA-NaOH 2 mM pH 8.0 buffer (IRF-7, RIG-I). After blocking endogenous peroxidases and non-specific binding, the sections were incubated at 4°C overnight with one of the following antibodies at the indicated dilutions: polyclonal anti-IFN-β (1:750; sc-20107, Santa Cruz Biotechnology Inc, Santa Cruz, USA), monoclonal anti-IRF-7 (1:200; sc-74472, Santa Cruz Biotechnology Inc, Santa Cruz, USA), polyclonal anti-RIG-I (1:600; sc-98911, Santa Cruz Biotechnology Inc, Santa Cruz, USA), polyclonal anti-MDA-5 (1:500; ab69983, Abcam, Cambridge, UK). Negative controls were obtained by incubating a slide per staining run with rabbit IgG (1:500; ab37415, Abcam, Cambridge, UK) or mouse IgG (1:200 ab37355, Abcam, Cambridge, UK) isotype controls. Immunostaining was performed using the ABC immunoperoxidase method (Vectastain Elite ABC kit; Vector Laboratories, Burlingame, USA) with a DAB reaction. The slides were then counterstained in hematoxylin, dehydrated, and mounted.

**Scoring of Immunolabeling**

Immunostaining was scored by two independent observers (J.G.V. and J.O.G.), without knowledge of the histories, outcome and other clinicopathologic parameters of the patients. Labeling semiquantitation was performed following previously published scoring criteria (van Diest, 1997; Cao, 2007; Charafe-Jauffret, 2004) which are based on the dominant staining intensity and the extent of immunoreactivity. In our design, the intensity (I) was defined in six categories (0, negative; 1, trace; 2, weak; 3, intermediate; 4, strong; 5, very strong/saturated). The extent (P) of immunostaining was determined as percentage (0% to 100%) of positive cells.

The immunostaining scores (IS) were obtained by multiplying the intensity of staining (I) by the percentage of positive cells (P) divided by 100 (IS = I x P/100). Resulting scores ranged from 0 to 5.

At least four microscopic fields per tissue compartment and patient were scored by means of an Olympus CH2 light microscope equipped with a calibrated NE35 eyepiece graticule (Electron Microscopy Sciences, Hatfield, PA, USA). By using a x25 or x40 objective, each field encompassed 0.1849 (430 μm x 430 μm) or 0.0625 (250 μm x 250 μm) square millimeters, respectively.

**DNA and RNA extraction and quantification**

Genomic DNA was obtained from tissue samples by proteinase K digestion followed by phenol-clorophorm extraction and ethanol precipitation, and finally, it was resuspended into water, as previously described (Blin, 1976). Extracted DNA was purified using a commercial spin column procedure according to the supplier's protocol (AllPrep DNA mini spin column, QIAGEN, Hilden, Germany). Total RNA from tissue samples was isolated using the AllPrep DNA/RNA Mini Kit (QIAGEN). Quantity and quality of extracted DNA and RNA was assessed using NanoDrop 2000c spectrophotometer (Thermo Fisher Scientific, DE, USA).

**Quantitative real-time PCR**

Total RNA from tissue samples was isolated using the AllPrep DNA/RNA Mini Kit (QIAGEN). Quantity and quality of extracted RNA was assessed using NanoDrop 2000c spectrophotometer (Thermo Fisher Scientific, DE, USA).

Expression of *IRF7, MDA5, RIG-I* and *IFN-β* genes in tissue samples was also examined by qRT-PCR. Total isolated RNA (1.5 μg) was reversely transcribed into complementary DNA (cDNA) using the First Strand cDNA Synthesis Kit (Fermentas, Thermo Scientific, Chicago, IL, USA) and combined with TaqMan 2X Universal PCR Master Mix (Applied Biosystems, Foster City, CA, USA) according to the supplier's instructions. For the analysis of mRNA levels, qRT-PCR was performed using commercially available primer and probe sets (inventoried TaqMan Gene Expression Assays) purchased from Applied Biosystems. qRT-PCR reactions were carried out using the ABI PRISM 7900HT Real-Time PCR System (Applied Biosystems). Thermal profile consisted in 2 min at 50C and 10 min at 95C, followed by 40 cycles of 15 sec at 95C and 1 min at 60C. Data were collected using the SDS v2.1 software (Applied Biosystems) and analyzed by the comparative Ct (2^-ΔΔCt^) quantification method using the Expression Suite v1.0 software (Applied Biosystems). The relative expression levels of *IRF7, MDA5, RIG-I* and *IFN-β* genes were determined using 18S mRNA as an endogenous control for normalization. Results are expressed as the mean of the relative quantification (RQ) of the tested transcripts. Results were obtained from 3 independent experiments performed using 1 μg of cDNA, all with triplicate measurements. No signal was detected in non-template controls.

**Statistical Analysis**

The immunohistochemistry scores from each observer were compared for interobserver reliability by use of a two-way random effect model with absolute agreement definition. Differences among groups were analyzed using the Kruskal-Wallis test for continuous variables. When differences were significant, the Kruskal-Wallis test was followed by the Mann-Whitney U test was used for comparison between groups with Bonferroni correction for multiple comparisons. When in the pairwise comparisons it was found that there were significant differences between all four groups, the data were grouped into a Control group and a COPD group, analyzing the differences through the Mann Whitney U test and incorporating that difference into the histograms. The chi-square test (or the Fisher exact test when one of the expected effects was less than 5) was used for qualitative variables. All comparisons were two-sided and the significance level was set at p < 0.05. A simple regression analysis was performed to assess the relationship between pairs of selected parameters from immunoscores, physiological data and emphysema estimators. All analyses were performed by using Statgraphics Centurion XV (StatPoint Inc., Virginia, USA) software.

**References**

Blin N, Stafford DW. A general method for isolation of high molecular weight DNA from eukaryotes. Nucl Acids Res. 1976;3(9):2303-8.

Cao W, Zhang B, Liu Y, Li H, Zhang S, Fu L, et al. High-level SLP-2 expression and HER-2/neu protein expression are associated with decreased breast cancer patient survival. Am J Clin Pathol. 2007;128(3):430-6. PubMed PMID: 17709317.

Charafe-Jauffret E, Tarpin C, Bardou VJ, Bertucci F, Ginestier C, Braud AC, et al. Immunophenotypic analysis of inflammatory breast cancers: identification of an 'inflammatory signature'. J Pathol. 2004;202(3):265-73. PubMed PMID: 14991891.

Hsia CC, Hyde DM, Ochs M, Weibel ER. An official research policy statement of the American Thoracic Society/European Respiratory Society: standards for quantitative assessment of lung structure. Am J Respir Crit Care Med. 2010;181(4):394-418. PubMed PMID: 20130146.

Nyengaard JR, Gundersen HJG. Sampling for stereology in lungs. Eur Respir Rev. 2006;15(101):107-14.

Thurlbeck WM, Dunnill MS, Hartung W, Heard BE, Heppleston AG, Ryder RC. A comparison of three methods of measuring emphysema. Hum Pathol. 1970;1(2):215-26. PubMed PMID: 5521724.

van Diest PJ, van Dam P, Henzen-Logmans SC, Berns E, van der Burg ME, Green J, et al. A scoring system for immunohistochemical staining: consensus report of the task force for basic research of the EORTC-GCCG. European Organization for Research and Treatment of Cancer-Gynaecological Cancer Cooperative Group. J Clin Pathol 1997;50(10):801-4.
