# Supplementary material for: Correction: Deficient pulmonary IFN-β expression in COPD patients
Source: PLoS One. 2019 Jul 1;14(7):e0219349. doi: 10.1371/journal.pone.0219349 (PMC6602286; doi:10.1371/journal.pone.0219349)
Supplement: S2 File — (PDF) [file pone.0219349.s002.pdf]

PLOS ONE

RESEARCH ARTICLE

Deficient pulmonary IFN-β expression in COPD patients

José García-Valero[1]*, **Jordi Olloquequi**[1,2], **Juan F. Montes**[1], **Esther Rodríguez**[3], **Mireia Martín-Satué**[4,5], **Laura Texidó**[4], **Jaume Ferrer Sancho**[3]

1 Department of Cell Biology, Physiology and Immunology, Faculty of Biology, University of Barcelona, Barcelona, Spain, **2** Instituto de Ciencias Biomédicas, Universidad Autónoma de Chile, Talca, Chile, **3** Department of Pneumology, Vall d'Hebron University Hospital, Universitat Autònoma de Barcelona and CIBER de Enfermedades Respiratorias (CIBERES), Barcelona, Spain, **4** Department of Pathology and Experimental Therapeutics, Faculty of Medicine, University of Barcelona, Barcelona, Spain, **5** Institut d'Investigació Biomèdica de Bellvitge (IDIBELL), Barcelona, Spain

* jgarcia@ub.edu

Check for updates

OPEN ACCESS

Citation: García-Valero J, Olloquequi J, Montes JF, Rodríguez E, Martín-Satué M, Texidó L, et al. (2019) Deficient pulmonary IFN-β expression in COPD patients. PLoS ONE 14(6): e0217803. https://doi.org/10.1371/journal.pone.0217803

Editor: Aran Singanayagam, Imperial College London, UNITED KINGDOM

Received: March 5, 2019

Accepted: May 18, 2019

Published: June 6, 2019

Copyright: © 2019 García-Valero et al. This is an open access article distributed under the terms of the Creative Commons Attribution License, which permits unrestricted use, distribution, and reproduction in any medium, provided the original author and source are credited.

Data Availability Statement: All relevant data are within the manuscript and its Supporting Information files.

Funding: This study was supported by the grants received by JFS from the Health Research Fund (Madrid, Spain; FIS 04/0635) and the Sociedad Española de Pneumologia (SEPAR 165|2012). None of the funders played any role in the study design, data collection and analysis, decision to publish, or preparation of the manuscript.

Competing interests: The authors have declared that no competing interests exist.

Abstract

COPD patients are prone to acute infectious exacerbations that impair their quality of life and hamper prognosis. The purpose of the present study was to investigate the *in situ* IFN-β response in the lungs of stable COPD and non-COPD patients. Lung samples from 70 subjects (9 control never smokers, 19 control smokers without COPD, 21 patients with moderate COPD and 21 patients with very severe COPD) were studied for the expression of IFN-β, its main transcription factor, IRF-7, and two products of its autocrine function, namely RIG-I and MDA-5, by immunohistochemical techniques and quantitative real-time PCR. IFN-β, IRF-7, RIG-I and MDA-5 were widely detected in most lung cell types. In epithelial tissues and alveolar macrophages, IFN-β and IRF-7 labeling scores were decreased up to 65% and 74%, respectively, for COPD patients, paralleling an analogous reduction (43% and 65%, respectively) in the amount of their lung mRNA. Moreover, this decreased production of IFN-β in COPD patients correlated with a similar decrease in the expression of RIG-I and MDA-5, two essential members of the innate immune system. Our study indicates that most lung cells from stable COPD patients show a constitutive decreased expression of IFN-β, IRF-7, RIG-I and MDA-5, suggesting that this deficiency is the main cause of their acute viral exacerbations.

Introduction

Current understanding about what role risk factors, other than tobacco, play in the pathogenesis of Chronic Obstructive Pulmonary Disease (COPD) is still incomplete [1–3]. In this regard, recent results clearly emphasize the importance of respiratory infections, the imbalance of lung microbiome, and host-microorganism interactions in fueling the progression of chronic lung diseases, including asthma and COPD [4–6].

Although there have been contradictory results about the involvement of lung bacterial microbiota on COPD pathogenesis [7], viral infections are considered a significant cause of

exacerbations in COPD patients [8–10]. Moreover, acute viral infections, that have even been experimentally tested in humans [11], are considered key events in COPD progression, boosting the underlying chronic inflammation [12] by increasing viral load [13], activating latent infections [12], or impairing the innate immune response [14].

Type I interferons (IFNs) induce cell-intrinsic antimicrobial states in infected and neighboring cells that limit the spread of infectious agents. Also, they promote antigen presentation and natural killer cell functions, mainly activating the adaptive immune system while restraining pro-inflammatory pathways and cytokine production [15]. Additionally, type I IFNs, including IFN-β, are constitutively secreted in low amounts by many tissues to maintain basal expression of IFN-inducible signaling intermediates (STAT1/2, IRF7/9/5), thereby priming the cells for future responses [16–18].

Two *in vitro* studies using bronchial cells from patients with COPD showed conflicting results after being infected with rhinoviruses. While one showed an increase in the production of pro-inflammatory cytokines and interferons [19], a more recent one indicated an impaired induction of IFN-β expression [20]. Nevertheless, a deficient response is more consistent with other studies, such as that carried out by Mallia *et al* [11], which demonstrated that IFN production was impaired in experimentally infected bronchoalveolar lavage cells from subjects with COPD. It is also important to note that the deficit in this cornerstone antiviral cytokine would affect the stable, as well as the exacerbated patients, since Hilzendeger *et al* [21] demonstrated that these subjects showed a reduced expression of some interferon-stimulated genes (ISG) in induced sputum.

From the abovementioned evidence, we hypothesize that COPD patients have an impairment in the expression of IFN, resulting in an increased susceptibility to infection. In this regard, the purpose of this study was to analyze the cell type expression of IFN-beta, its main transcription factor (IRF-7), and two ISG products (RIG-I, retinoic acid-inducible gene 1 protein and MDA-5, melanoma differentiation-associated gene 5) in lung samples from COPD and non-COPD patients.

Materials and methods

Subjects

The study was approved by the ethics committee of the Vall d'Hebron University Hospital, Barcelona, Spain (certificate of ethical approval PI 040635), and all subjects provided written informed consent. Seventy subjects, recruited between 2005 and 2010, who underwent lung resection for non-obstructive peripheral lung tumors or were subjected to lung transplantation for very severe COPD were studied (Table 1). Non-COPD groups were recruited consecutively among patients undergoing lung surgery for lung cancer. Patients with other respiratory conditions than lung cancer were excluded. COPD diagnosis was based on spirometry according to ERS recommendations and non-COPD patients were divided into smokers (current or ex-smokers), and non-smokers. We evaluated anti-viral IFN-β response in lung samples from 70 subjects (9 control never smokers, 19 control smokers without COPD, 21 patients with moderate COPD and 21 patients with very severe COPD) by immunohistochemical techniques and quantitative real-time PCR.

Table 1 shows the number of subjects, demographic and smoking characteristics, spirometry variables and macroscopic emphysema degree in each group.

Tissue processing for Microscopy and Immunohistochemistry

The resected lungs or lobes obtained in surgery were immediately fixed to grade the macroscopic severity of emphysema. Immunohistochemistry was performed according to standard

Table 1. Demographic and clinical outcomes in the four groups of patients.

	Never-smokers n = 9	Smokers without COPD n = 19	Moderate COPD n = 21	Very severe COPD n = 21	p-for-trend
Age (years), (SD)	62 (14)	60 (11)	64 (8)	55 (5)	0.121
Sex, male vs. female	1/8	15/4	21/0	18/3	
BMI (Kg/m^2), (SD)	27 (9)	26 (4)	24 (3)	26 (5)	0.385
Pack-years, (SD)	0 (0)	50 (24)	71 (28)	48 (22)	0.792
Dyspnea score (mMRC >2), n (%)	0 (0)	1 (5)	0 (0)	21 (100)	<0.001
Chronic bronchitis symptoms, n (%)	0 (0)	3 (16)	6 (29)	13 (62)	<0.001
Any exacerbations in the last year, n (%)	0 (0)	0 (19)	10 (48)	17 (81)	<0.001
N˚ of patients treated with ICS, n (%)	0 (0)	1 (5)	8 (38)	20 (95)	<0.001
N˚ of patients with oxygen, n (%)	0 (0)	0 (0)	0 (0)	16 (76)	<0.001
FEV_1 post-bronchodilator (% pred), (SD)	99 (24)	85 (11)	69 (17)	20 (4)	<0.001
FEV_1/FVC post-bronchodilator (% pred), (SD)	80 (8)	78 (6)	59 (8)	36 (8)	<0.001
MED % (SD)	1.7 (3)	13 (16)	29 (17)[a]	62 (21)	<0.001
LM[b] (:m) (SD)	196 (93)	255 (132)	357 (226)	467 (283)	0.002

SD: Standard Deviation; BMI: Body Mass Index; mMRC: Modified Medical Research Council Dyspnea Scale; ICS: Inhaled corticosteroids; MED: Macroscopic Emphysema Degree; LM: Mean airspace chord length.

[a]Macroscopic emphysema degree non available in one patient.

[b]Patients without data of LM available. 1 never smoker, 1 smoker without COPD and 1 moderate COPD.

https://doi.org/10.1371/journal.pone.0217803.t001

procedures. Briefly, deparaffinized sections were processed at 121˚C in a 2100 Retriever (Pick-Cell Laboratories, Leiden, Netherland) in Tris-HCl 10 mM-EDTA 0.5 mM pH 9.0 buffer (IFN-β, MDA-5), or in EDTA-NaOH 2 mM pH 8.0 buffer (IRF-7, RIG-I). After blocking endogenous peroxidases and non-specific binding, the sections were incubated at 4˚C overnight with one of the following antibodies at the indicated dilutions: polyclonal anti-IFN-β (1:750; sc-20107, Santa Cruz Biotechnology Inc, Santa Cruz, USA), monoclonal anti-IRF-7 (1:200; sc-74472, Santa Cruz Biotechnology Inc, Santa Cruz, USA), polyclonal anti-RIG-I (1:600; sc-98911, Santa Cruz Biotechnology Inc, Santa Cruz, USA), polyclonal anti-MDA-5 (1:500; ab69983, Abcam, Cambridge, UK). Negative controls were obtained by incubating a slide per staining run with rabbit IgG (1:500; ab37415, Abcam, Cambridge, UK) or mouse IgG (1:200 ab37355, Abcam, Cambridge, UK) isotype controls. Immunostaining was performed using the ABC immunoperoxidase method (Vectastain Elite ABC kit; Vector Laboratories, Burlingame, USA) with a DAB reaction. The slides were then counterstained in hematoxylin, dehydrated, and mounted.

Scoring of Immunolabeling

Immunostaining was scored by two independent observers (JGV and JO), without knowledge of the histories, outcome and other clinicopathologic parameters of the patients. Labeling semiquantitation was performed following usual scoring criteria based on the dominant staining intensity and the extent of immunoreactivity. In our design, the intensity (I) was defined in six categories (0, negative; 1, trace; 2, weak; 3, intermediate; 4, strong; 5, very strong/saturated). The extent (P) of immunostaining was determined as percentage (0% to 100%) of positive cells.

The immunostaining scores (IS) were obtained by multiplying the intensity of staining (I) by the percentage of positive cells (P) divided by 100 (IS = I x P/100). Resulting scores ranged from 0 to 5.

At least four microscopic fields per tissue compartment and patient were scored by means of an Olympus CH2 light microscope equipped with a calibrated NE35 eyepiece graticule (Electron Microscopy Sciences, Hatfield, PA, USA). By using a x25 or x40 objective, each field encompassed 0.1849 (430 μm x 430 μm) or 0.0625 (250 μm x 250 μm) square millimeters, respectively.

Quantitative real-time PCR

Total RNA from tissue samples was isolated using the AllPrep DNA/RNA Mini Kit (QIAGEN, Hilden, Germany). Expression of *IRF7*, *IFNB*, *MDA5* and *RIG-I* genes in tissue samples was examined by qRT-PCR. After reverse transcription of mRNA into complementary DNA (cDNA), qRT-PCR was performed using commercially available primer and probe sets (inventoried TaqMan Gene Expression Assays) purchased from Applied Biosystems (Foster City, USA). After carrying out qRT-PCR reactions, data were analyzed by the comparative Ct ($2^{-\Delta\Delta Ct}$) quantification method. Relative expression levels of *IRF7*, *IFNB*, *MDA5* and *RIG-I* genes were determined using *18S* mRNA as an endogenous control. Results from 3 independent experiments were expressed as the mean of the relative quantification (RQ) of the tested transcripts. No signal was detected in non-template controls.

Statistical analysis

Differences among groups were analyzed using the Kruskal-Wallis test for continuous variables. When differences were significant, the Kruskal-Wallis test was followed by the Mann-Whitney U test for comparison between groups with Bonferroni correction for multiple comparisons. When in the pairwise comparisons it was found that there were significant differences between all four groups, the data were grouped into a Control group and a COPD group, analyzing the differences through the Mann Whitney U test and incorporating that difference into the histograms. The chi-square test (or the Fisher exact test when one of the expected effects was less than 5) was used for qualitative variables. A simple regression analysis was performed to assess the relationship between pairs of selected parameters from immunoscores, physiological data and emphysema estimators. All analyses were performed by using Statgraphics Centurion XV (StatPoint Technologies Inc., Virginia, USA) software.

Results

Subjects

The four clinical groups were similar with regard to age, with a predominance of males, except in the group of never smoker patients (Table 1). No significant difference was found in pack-years among smokers with and without COPD. As expected, respiratory symptoms, previous exacerbations and macroscopic emphysema degree (MED, %) were increased and spirometry variables were decreased in COPD patients, especially in those with very severe COPD. A higher use of inhaled corticosteroids (ICS) and home oxygen therapy was also present in COPD groups.

Peripheral lung tissue from COPD patients shows a reduced IFN-β expression

All samples analyzed by immunohistochemistry were positive for IFN-β, although epithelial tissues, and alveolar macrophages from stable COPD patients (Figs 1 and 2) showed significantly lower labeling scores than those from the control groups (Table 2). Thus, IFN-β immunostaining score in respiratory epithelium was decreased up to 40% for moderate COPD and

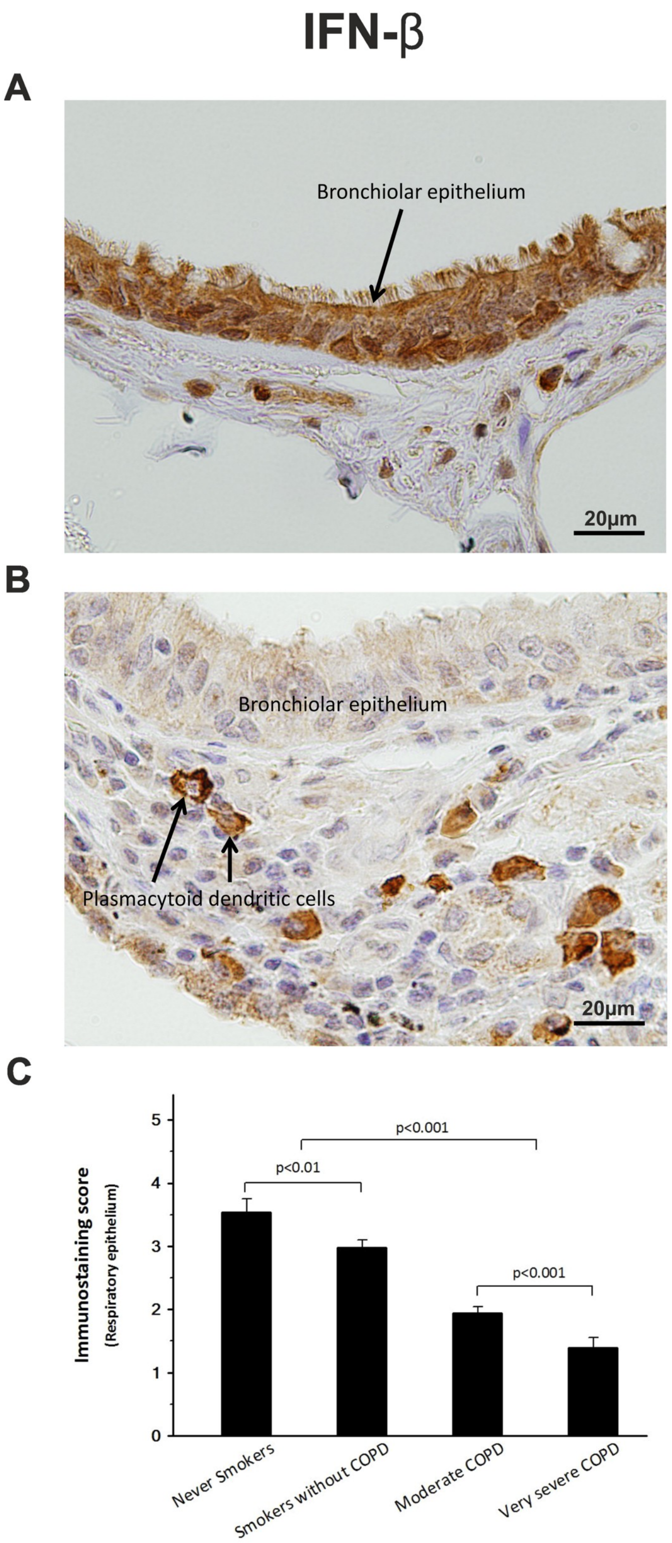

Fig 1. IFN-β expression. Representative pictures of peripheral lung sections from never smokers (A) and very severe COPD patients (B) stained with an antibody against IFN-β. High expression of IFN-β in the never smokers group (A) contrasts to the weak expression shown by very severe COPD patients (B), except for plasmacytoid dendritic cells. (C) IFN-β expression is decreased in the respiratory epithelium of COPD patient groups. The upper bar indicates the differences between non-COPD patients and COPD patients.

https://doi.org/10.1371/journal.pone.0217803.g001

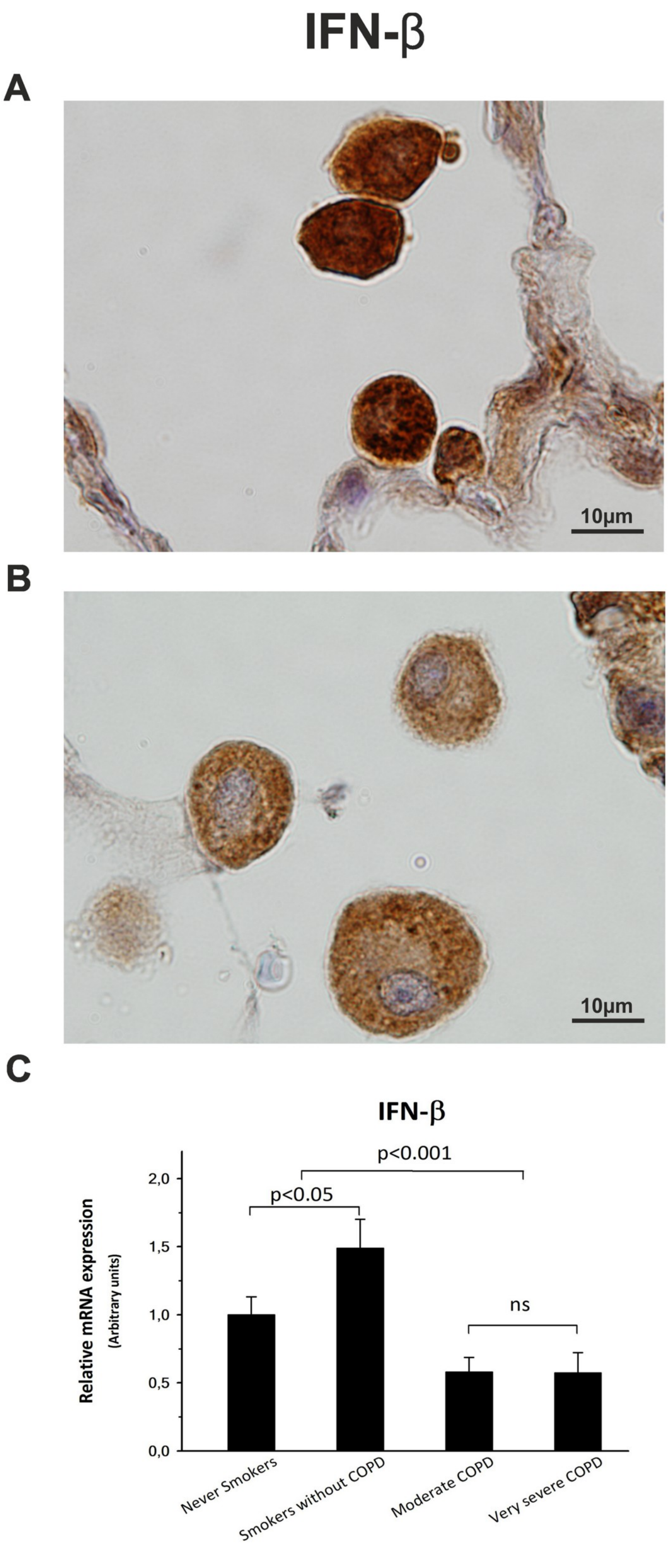

Fig 2. IFN-β expression. Representative pictures of immunohistochemistry for IFN-β in alveolar macrophages from a never smoker patient (A) and from a very severe COPD patient (B). (C) Representative histogram showing the IFN-β mRNA expression, estimated by qPCR, among patient groups. The upper bar indicates the differences between non-COPD patients and COPD patients.

https://doi.org/10.1371/journal.pone.0217803.g002

Table 2. IHC immunoscores in several cell types (mean ∀ se).

	Never smokers	Non-COPD smokers	Moderate COPD	Severe COPD
**IFN-β** (Respiratory epithelium)	3.54 ± 0.21	2.97 ± 0.13 (p<0.001)	1.94 ± 0.10 (p<0.001)	1.39 ± 0.17 (p<0.001)
**IFN-β** (Alveolar macrophages)	4.12 ± 0.12	3.21 ± 0.14 (p<0.01)	2.43 ± 0.11 (p<0.001)	1.98 ± 0.14 (p<0.001)
**IFN-β** (Plasmacytoid dendritic cells)	3.77 ± 0.30	3.32 ±0.22 (p<0.05)	3.58 ± 0.12 (p<0.03)	3.79 ± 0.17 (ns)
**IRF-7** (Respiratory epithelium-cyt-)	4.43 ± 0.16	4.05 ± 0.19 (ns)	1.69 ± 0.11 (p<0.001)	1.16 ± 0.12 (p<0.001)
**IRF-7** (Respiratory epithelium-nuc-)	4.21 ± 0.46	2.87 ± 0.36 (p<0.05)	0.70 ± 0.21 (p<0.001)	0.47 ± 0.23 (p<0.001)
**IRF-7** (Alveolar macrophages-cyt-)	3.78 ± 0.36	3.06 ± 0.28 (p<0.05)	1.33 ± 0.27 (p<0.001)	1.04 ± 0.24 (p<0.001)
**IRF-7** (Alveolar macrophages-nuc-)	4.24 ± 0.31	3.20 ± 0.32 (p<0.05)	0.72 ± 0.20 (p<0.001)	0.83 ± 0.22 (p<0.001)
**IRF-7** (Plasmacytoid dendritic cells-cyt-)	1.23 ± 0.40	1.33 ± 0.35 (ns)	1.81 ± 0.39 (ns)	1.62 ± 0.34 (ns)
**MDA-5** (Respiratory epithelium -cyt-)	4.26 ± 0.28	4.01 ± 0.39 (ns)	1.25 ± 0.22 (p<0.001)	0.49 ± 0.20 (p<0.001)
**RIG-I** (Respiratory epithelium -cyt-)	3.99 ± 0.13	3.39 ± 0.12 (p<0.05)	2.20 ± 0.10 (p<0.001)	1.53 ± 0.05 (p<0.001)

p: p-value compared with the Never smokers group; ns: not significant; nuc: nucleus; cyt: cytoplasm

https://doi.org/10.1371/journal.pone.0217803.t002

65% for very severe COPD, very similar to the decline observed in alveolar macrophages (40% and 50%, respectively). Moreover, quantitative PCR analysis revealed that the decrease observed in the IFN-β immunoscore paralleled an analogous reduction up to 43% in the amount of lung mRNA (Fig 2C).

Conversely, no significant differences were found among COPD and non-COPD patient groups in the IFN-β expression of the plasmacytoid cells (Fig 1B, Table 2).

Decrease in IFN-β expression correlates with alterations in the IRF-7 signaling pathway

Immunostaining scores for IRF-7, the main regulator of type I interferon (type I IFN) expression, were also significantly lower in COPD groups, showing a decrease of 60% and 75% in the cytoplasm of both respiratory epithelium and alveolar macrophages from moderate and very severe COPD patients, respectively (Figs 3 and 4, Table 2).

A further reduction in the IRF-7 signaling of COPD patients was due to a decrease of 80–90%, depending on the cell type, in its nuclear translocation. Immunostaining scores (Table 2) for nuclei of respiratory epithelium and alveolar macrophages (Figs 3 and 4) illustrate the weakening in the IRF-7 pathway. Moreover, there was a significant correlation ($\rho = 0.62$, $p < 0.001$) between nuclear IRF-7 and IFN-β expression.

Quantitative PCR showed that not only the protein expression, but also the amount of mRNA for *IRF7* gene, was significantly lower (25–54%) in COPD patients than control subjects (Fig 4C).

COPD patients show mild expression of IFN-inducible viral sensors MDA-5 and RIG-I

Immunostaining scores for MDA-5, were also significantly lower in COPD groups, showing a decrease of 70% and 88% in the cytoplasm of respiratory epithelium cells from moderate and very severe COPD patients, respectively (Fig 5, Table 2).

Similarly, immunostaining scores for RIG-I, were significantly lower in COPD groups, showing a decrease of 45% and 62% in the cytoplasm of respiratory epithelium cells from moderate and very severe COPD patients, respectively (Fig 6, Table 2).

Results from qPCR indicate that MDA-5 and RIG-I mRNA expression was reduced by up to 60% among COPD patients compared with control subjects (Figs 5D and 6D).

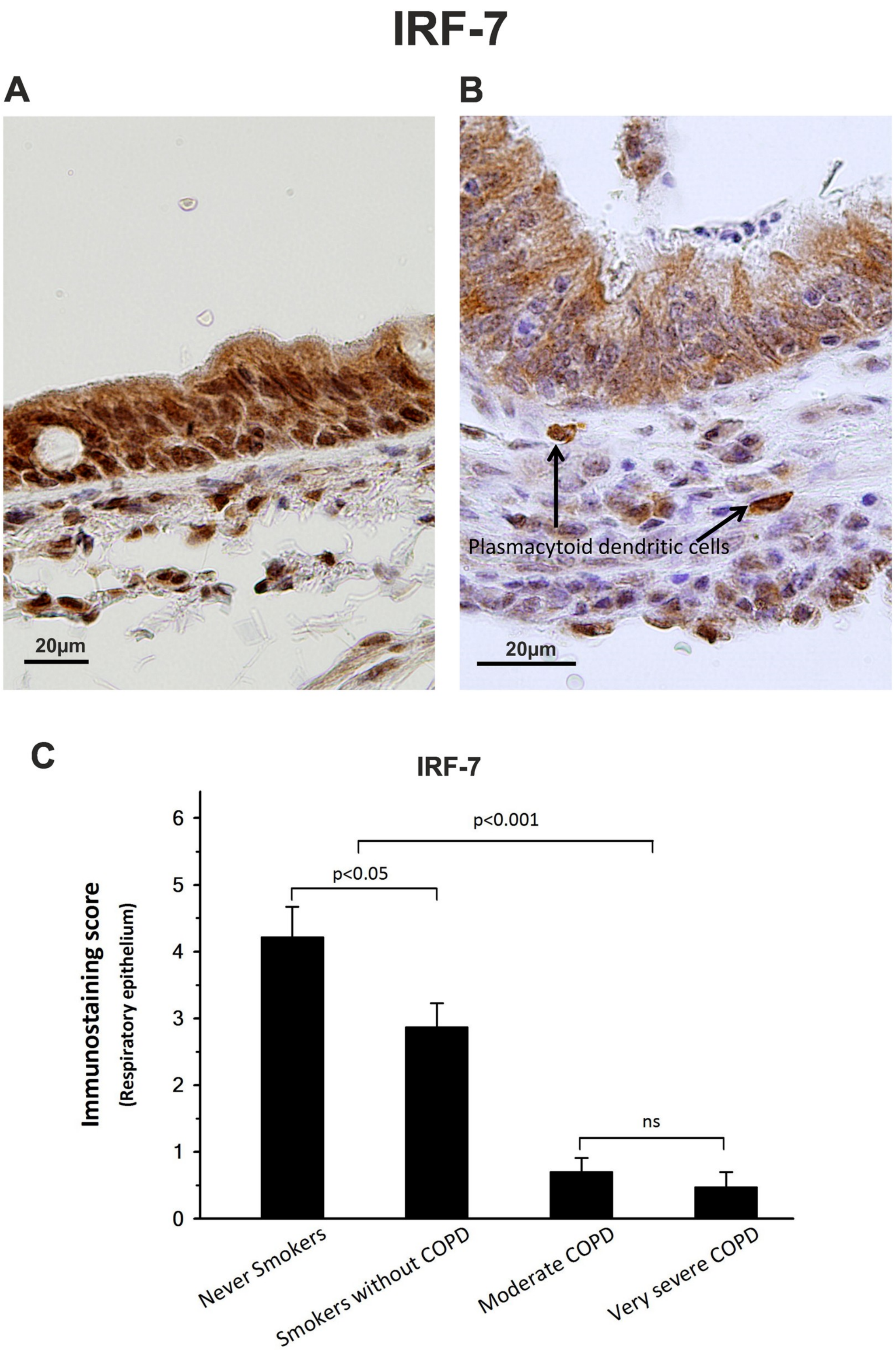

Fig 3. IRF-7 expression. Immunolocalization of IRF-7 in peripheral lung sections from never smokers (A) and very severe COPD patients (B). High nuclear expression of IRF-7 in control groups (A) contrasts with the poor IRF-7 expression and low nuclear translocation in COPD groups (B), with the exception of the plasmacytoid dendritic cells. (C) Histogram summarize the nuclear translocation of IRF-7 in the respiratory epithelium. The upper bar indicates the differences between non-COPD patients and COPD patients.

https://doi.org/10.1371/journal.pone.0217803.g003

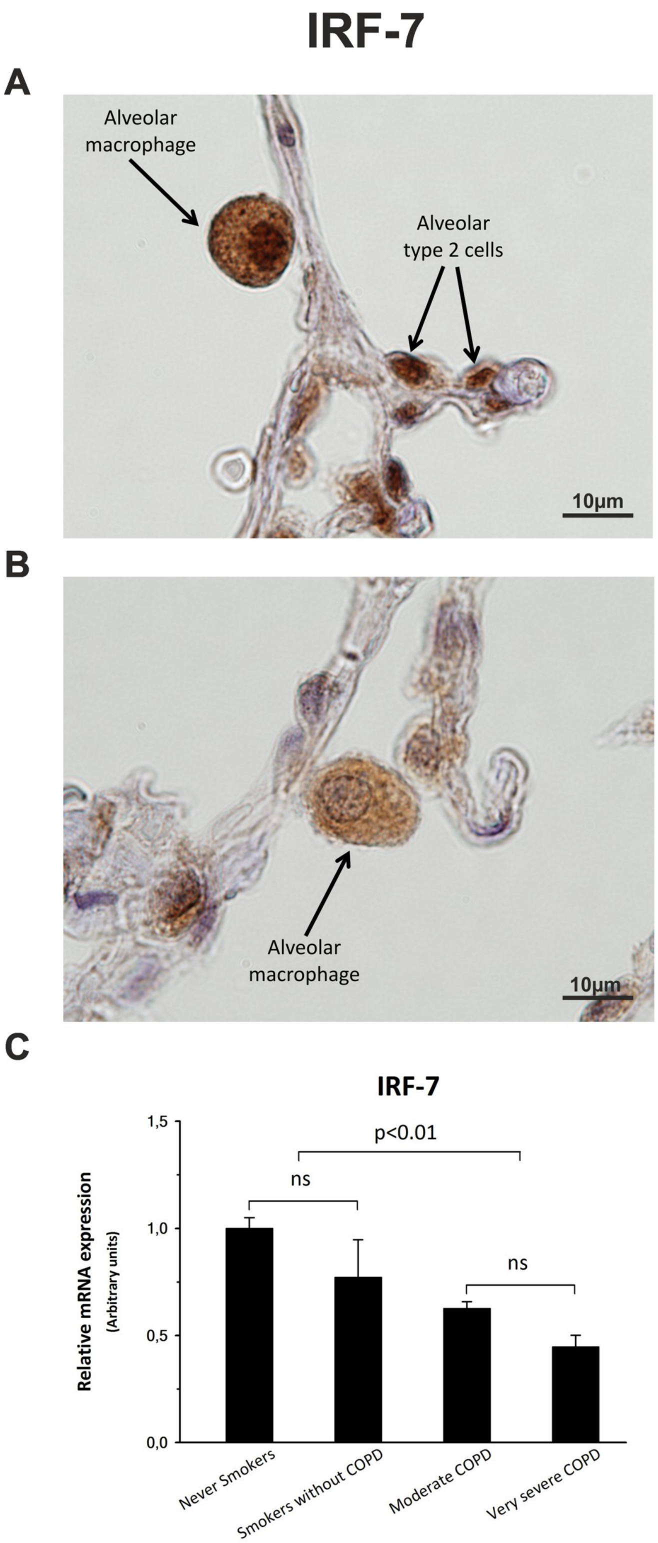

Fig 4. IRF-7 expression. Cytoplasmic immunolocalization and nuclear translocation in alveolar macrophages from a never smoker patient (A) and from a very severe COPD patient (B). (C) Representative histogram showing the IRF-7 mRNA expression, estimated by qPCR, among patient groups. The upper bar indicates the differences between non-COPD patients and COPD patients.

https://doi.org/10.1371/journal.pone.0217803.g004

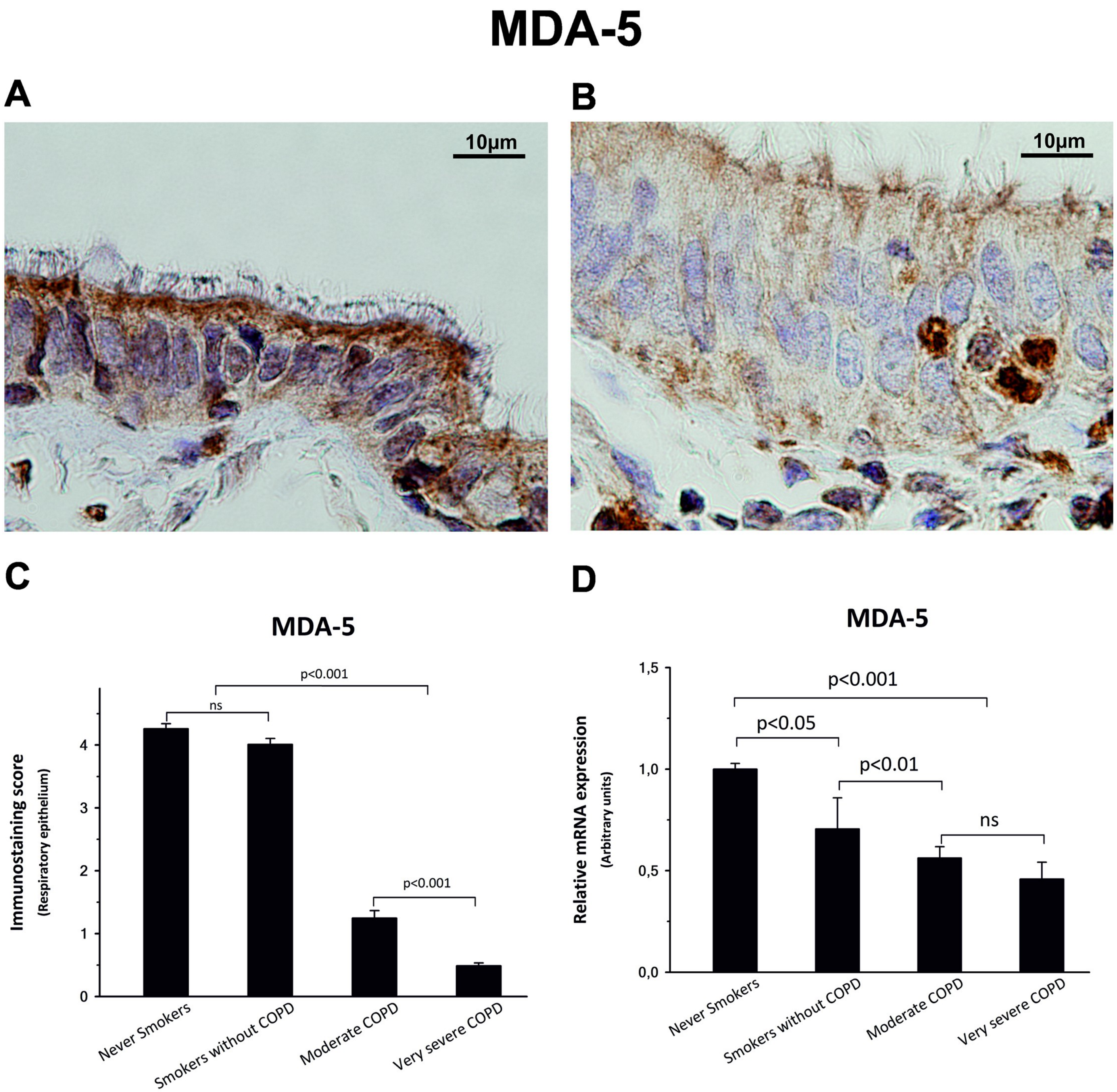

Fig 5. MDA-5 expression. Immunolocalization of MDA-5 in peripheral lung sections from never smokers (A) and very severe COPD patients (B). (C) Immunostaining score of respiratory epithelium was significantly increased in non-COPD patients, especially in apical compartment, when compared with COPD patients. (D) mRNA showed a similar expression pattern. The upper bar indicates the differences between non-COPD patients and COPD patients.

https://doi.org/10.1371/journal.pone.0217803.g005

Discussion

IFNs are cytokines expressed by most cells that regulate many different biologic functions, including the inflammatory and immune responses [22]. Type I IFN-β, playing a critical role

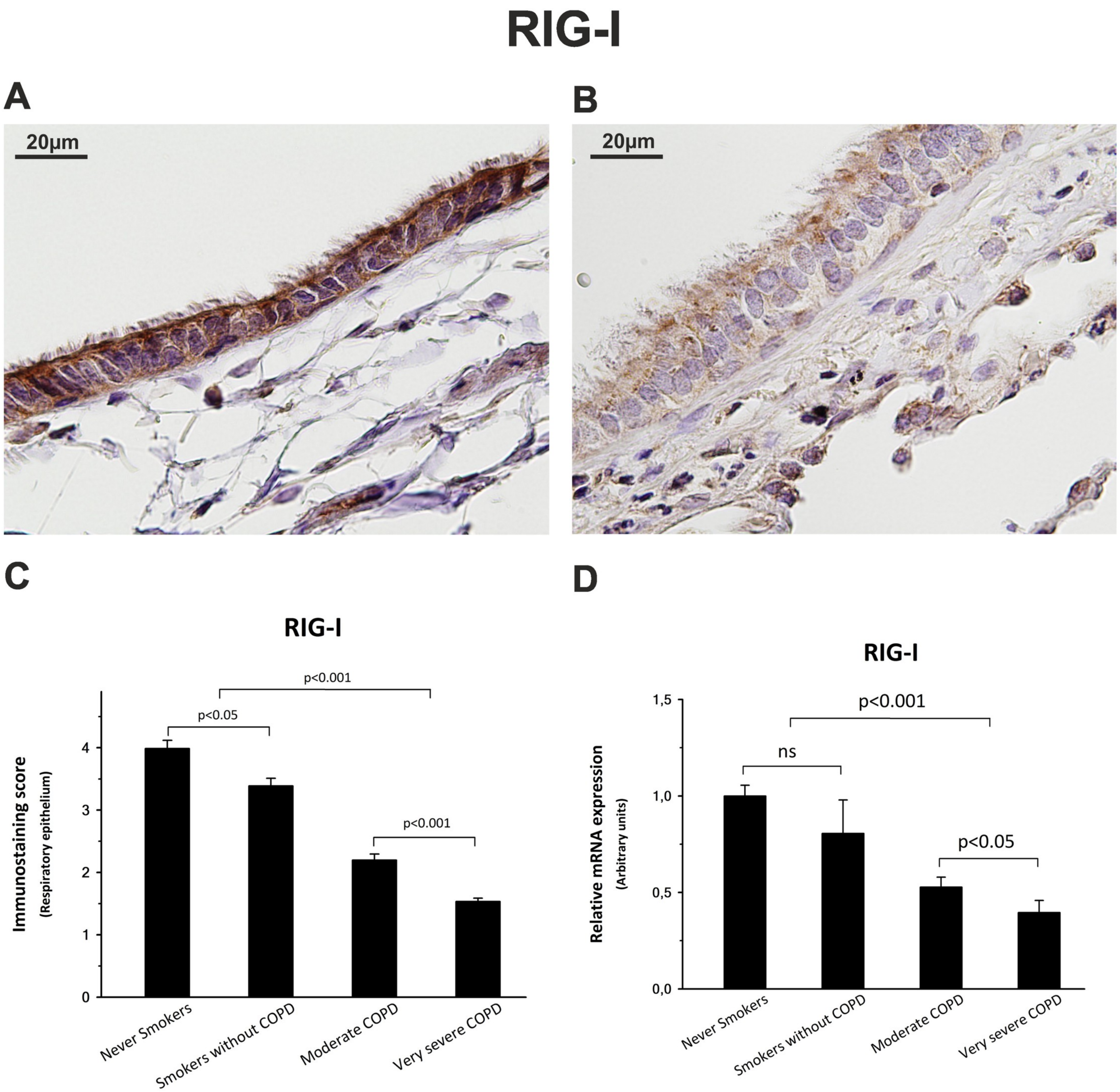

Fig 6. RIG-I expression. RIG-I showed a localization pattern very similar to MDA-5 after the immunostaining of lung sections from never smokers (A) and very severe COPD patients (B). Additionally, non-COPD and COPD patients showed similar significant differences in both the immunostaining score (C) of respiratory epithelium and the mRNA expression (D). The upper bar indicates the differences between non-COPD patients and COPD patients.

https://doi.org/10.1371/journal.pone.0217803.g006

in the regulation of innate immunity, is secreted abundantly in response to viral infection in nearly all cell types. Recently, it has been demonstrated [17] that IFN-β is also secreted constitutively in low amounts to induce a local state of alertness which assures rapid and efficient antiviral responses.

The previous results regarding type I interferon expression in COPD patients are contradictory, since while one of them shows an increase in the expression of cytokines and interferons [19], others show a decrease [11, 20–21]. Probably, some of the differences observed in these previous studies can be explained by there having been obtained from sputum or cell cultures (either from brushed airway epithelium or from bronchoalveolar lavage), but none of them has been directly observed in the tissue of patients.

The most notable result of our study was the evidence that IFN-β expression was, depending on cell type, 40–65% lower in lung cells from stable COPD patients, indicating a possible impairment in their local immune response. That could explain the greater likelihood of suffering exacerbations, which characteristically affect these subjects. Since all these patients were smokers, we used data from our control group of smokers without COPD to determine the effect of smoking on the decline in the observed IFN-β production. The results obtained in this group, in agreement with those obtained previously [23], indicated that smoking only accounted for 16% of the IFN-β decrease observed in COPD patients. Therefore, the reduction of lung IFN-β expression in stable COPD patients is probably due to the malfunction of signaling pathways controling its production.

The effect of corticosteroids on IFN activity has been recently studied. Glucocorticoids are known to bind with receptor-interacting protein 1 (GRIP-1) and suppress ISG expression [15]. Inhaled corticosteroids have been shown to impair the production of IFN-I in response to a viral infection in mice [24] and also in cells from asthmatic subjects treated with high doses of glucocorticoids [25]. In contrast, non-infected epithelial cells treated with glucocorticoids did not show a decrease in type I IFN production [26]. As far as we know, no data are available on the effect of ICS either on the lung antiviral response or on the constitutive type I IFN production in stable COPD patients. In our study, there were no differences between IFN-β epithelial cell and alveolar macrophage levels in moderate COPD patients who were or were not treated with ICS. Hence, the abovementioned absence of differences in IFN-β expression in our samples does not support an influence of corticosteroids on IFN production, but the real impact of this effect should be evaluated in further studies.

Given that the alveolar macrophages are an important source of type I IFN [27] and that the disease includes the destruction of the parenchyma, it could be inferred that at least a significant part of the decrease in the expression of IFN-β could be due to the loss of alveolar macrophages. However, the disease is characterized by the increase (up to 10 times) in the number of macrophages, both those infiltrated in the lung tissue and the alveolar macrophages [28–30]. Moreover, the increase in the number of macrophages is related with the severity of the disease [31].

Furthermore, lung production of type I IFN depends not only on alveolar macrophages, but also on other cells, such as epithelial cells, interstitial macrophages and, above all, plasmacytoid dendritic cells [32], which produce constitutively type I IFN.

Referring to transcription factors implicated in the expression of this crucial cytokine, only activated IRF-7 is essential [33], being considered the master regulator of type I IFN genes [34]. Therefore, the 62–74% reduction that we detected in the cytoplasmic expression of IRF-7 would explain by itself the poor production of IFN-β recorded in our COPD groups, as well as the reduced sputum expression of interferon stimulated genes recently observed in severe COPD patients [21]. The additional decrease of up to 90% observed in the nuclear translocation of IRF-7 not only strongly supports our hypothesis, but also indicates that this factor is deficiently imported to the nucleus of cells from COPD patients in order to activate *IFNB* gene transcription.

However, it is important to note that neighboring plasmacytoid dendritic cells, which constitutively secrete high levels of type I IFN [35], showed a similarly high expression in all

samples from both COPD and control groups. Therefore, the decrease in the expression of IFN-β in many, but not all, lung cells of COPD patients cannot be caused by an alteration in the single *IFNB* encoding gene [36], but rather by anomalous, probably epigenetic, regulation of this gene.

RIG-I and MDA-5, two well-known RIG-I-like cytosolic receptors (RLRs) for foreign nucleic acids [37–39], are an excellent guide to evaluate the IFN-β signaling pathway, since they are not only essential for the activation of IRF7, but are also products of the autocrine function of IFN-β and ISG activity. Interestingly, our results indicate that the significant decreases of up to 88% in protein expression and up to 60% in mRNA expression of these two important members of the innate immune system were related, as we expected, to the poor basal expression of IFN-β in COPD patients.

These results strongly suggest that COPD patients undergo a partial suppression, most likely of epigenetic origin, in the lung IFN-β gene expression. Moreover, this deficiency could be the cause of a negative control loop that would lead to a decrease in the production of MDA-5 and RIG-I, resulting in a poor IRF-7 activation and, ultimately, explaining the deficiency in IFN-β expression. Our data support the hypothesis that COPD patients have an impairment in their lung innate immunity and, consequently, are more susceptible to infections and thus more vulnerable to suffer exacerbations. We believe our results open a new window for a treatment with interferon for frequently exacerbated COPD patients.

Supporting information

S1 Text. Supplement for Materials and methods section.
(DOCX)

Acknowledgments

We thank the patients who participated in this study; Dr. Joaquim Majó and Dr. María Ángeles Montero (Anatomic Pathology Department, Vall d'Hebron University Hospital, Barcelona, Spain) for their valuable help in the histopathologic study of lung samples; Tania Martínez, for her technical support; Dr. Gloria Gannaway for her linguistic advice; and Dr. Marc Miravitlles and Dr. Joan Mª Vianney Blasi for their critical reading of the manuscript.

Author Contributions

Conceptualization: José García-Valero, Jaume Ferrer Sancho.

Data curation: Juan F. Montes, Esther Rodríguez, Laura Texidó.

Formal analysis: José García-Valero, Jordi Olloquequi, Esther Rodríguez, Laura Texidó.

Funding acquisition: Jaume Ferrer Sancho.

Investigation: José García-Valero, Jordi Olloquequi, Esther Rodríguez, Mireia Martín-Satué, Laura Texidó.

Methodology: José García-Valero, Jordi Olloquequi, Juan F. Montes, Esther Rodríguez, Mireia Martín-Satué.

Project administration: José García-Valero, Jordi Olloquequi, Jaume Ferrer Sancho.

Resources: Jordi Olloquequi, Esther Rodríguez.

Supervision: José García-Valero, Jaume Ferrer Sancho.

Validation: José García-Valero, Mireia Martín-Satué, Jaume Ferrer Sancho.

Visualization: José García-Valero, Jordi Olloquequi.

Writing – original draft: José García-Valero, Mireia Martín-Satué, Laura Texidó, Jaume Ferrer Sancho.

Writing – review & editing: José García-Valero, Jaume Ferrer Sancho.

References

1. GOLD. From the Global Strategy for the Diagnosis, Management and Prevention of COPD, 2019 Report. Global Initiative for Chronic Obstructive Lung Disease, Inc.; 2018.https://goldcopd.org/wp-content/uploads/2018/11/GOLD-2019-v1.7-FINAL-14Nov2018-WMS.pdf.
2. Postma DS, Bush A, van den Berge M. Risk factors and early origins of chronic obstructive pulmonary disease. The Lancet. 2015; 385(9971):899–909.
3. Hodgson DB, Saini G, Bolton CE, Steiner MC. Thorax in focus: chronic obstructive pulmonary disease. Thorax. 2012; 67(2):171. https://doi.org/10.1136/thoraxjnl-2011-201231 PMID: 22156780
4. Marsland BJ, Gollwitzer ES. Host-microorganism interactions in lung diseases. Nat Rev Immunol. 2014; 14(12):827–35. https://doi.org/10.1038/nri3769 PMID: 25421702.
5. Han MK, Huang YJ, LiPuma JJ, Boushey HA, Boucher RC, Cookson WO, et al. Significance of the microbiome in obstructive lung disease. Thorax. 2012; 67(5):456–63. https://doi.org/10.1136/thoraxjnl-2011-201183 PMID: 22318161
6. Leung JM, Tiew PY, Mac Aogain M, Budden KF, Yong VF, Thomas SS, et al. The role of acute and chronic respiratory colonization and infections in the pathogenesis of COPD. Respirology. 2017; 22(4):634–50. https://doi.org/10.1111/resp.13032 PMID: 28342288.
7. Sze MA, Utokaparch S, Elliott WM, Hogg JC, Hegele RG. Loss of GD1-positive Lactobacillus correlates with inflammation in human lungs with COPD. BMJ Open. 2015; 5(2):e006677. http://dx.doi.org/10.1136/bmjopen-2014-006677. PMID: 25652802.
8. Zwaans WA, Mallia P, van Winden ME, Rohde GG. The relevance of respiratory viral infections in the exacerbations of chronic obstructive pulmonary disease-a systematic review. J Clin Virol. 2014; 61(2):181–8. https://doi.org/10.1016/j.jcv.2014.06.025
9. George SN, Garcha DS, Mackay AJ, Patel ARC, Singh R, Sapsford RJ, et al. Human rhinovirus infection during naturally occurring COPD exacerbations. Eur Resp J. 2014; 44(1):87–96.
10. Seemungal T, Harper-Owen R, Bhowmik A, Moric I, Sanderson G, Message S, et al. Respiratory viruses, symptoms, and inflammatory markers in acute exacerbations and stable chronic obstructive pulmonary disease. Am J Respir Crit Care Med. 2001; 164(9):1618–23. https://doi.org/10.1164/ajrccm.164.9.2105011 PMID: 11719299.
11. Mallia P, Message SD, Gielen V, Contoli M, Gray K, Kebadze T, et al. Experimental rhinovirus infection as a human model of chronic obstructive pulmonary disease exacerbation. Am J Respir Crit Care Med. 2011; 183(6):734–42. https://doi.org/10.1164/rccm.201006-0833OC PMID: 20889904.
12. Hogg JC. Role of latent viral infections in chronic obstructive pulmonary disease and asthma. Am J Respir Crit Care Med. 2001; 164(10 Pt 2):S71–5. https://doi.org/10.1164/ajrccm.164.supplement_2.2106063 PMID: 11734471.
13. Utokaparch S, Sze MA, Gosselink JV, McDonough JE, Elliott WM, Hogg JC, et al. Respiratory viral detection and small airway inflammation in lung tissue of patients with stable, mild COPD. COPD. 2014; 11(2):197–203. https://doi.org/10.3109/15412555.2013.836166 PMID: 24088037.
14. Matsumoto K, Inoue H. Viral infections in asthma and COPD. Respir Investig. 2014; 52(2):92–100. https://doi.org/10.1016/j.resinv.2013.08.005 PMID: 24636264.
15. Ivashkiv LB, Donlin LT. Regulation of type I interferon responses. Nature reviews Immunology. 2014; 14(1):36–49. https://doi.org/10.1038/nri3581 PMID: 24362405
16. Gough DJ, Messina NL, Clarke CJP, Johnstone RW, Levy DE. Constitutive type I interferon modulates homeostatic balance through tonic signaling. Immunity. 2012; 36(2):166–74. https://doi.org/10.1016/j.immuni.2012.01.011 PMID: 22365663
17. Gough DJ, Messina NL, Hii L, Gould JA, Sabapathy K, Robertson AP, et al. Functional crosstalk between type I and II interferon through the regulated expression of STAT1. PLoS Biol. 2010; 8(4): e1000361. https://doi.org/10.1371/journal.pbio.1000361. PMID: 20436908.

18. Crotta S, Davidson S, Mahlakoiv T, Desmet CJ, Buckwalter MR, Albert ML, et al. Type I and Type III Interferons Drive Redundant Amplification Loops to Induce a Transcriptional Signature in Influenza-Infected Airway Epithelia. PLOS Pathogens. 2013; 9(11):e1003773. https://doi.org/10.1371/journal.ppat.1003773. PMID: 24278020
19. Schneider D, Ganesan S, Comstock AT, Meldrum CA, Mahidhara R, Goldsmith AM, et al. Increased cytokine response of rhinovirus-infected airway epithelial cells in chronic obstructive pulmonary disease. Am J Respir Crit Care Med. 2010; 182(3):332–40. https://doi.org/10.1164/rccm.200911-1673OC PMID: 20395558.
20. Hsu AC, Parsons K, Moheimani F, Knight DA, Hansbro PM, Fujita T, et al. Impaired Antiviral Stress Granule and IFN-beta Enhanceosome Formation Enhances Susceptibility to Influenza Infection in Chronic Obstructive Pulmonary Disease Epithelium. Am J Respir Cell Mol Biol. 2016; 55(1):117–27. https://doi.org/10.1165/rcmb.2015-0306OC
21. Hilzendeger C, da Silva J, Henket M, Schleich F, Corhay JL, Kebadze T, et al. Reduced sputum expression of interferon-stimulated genes in severe COPD. Int J Chron Obstruct Pulmon Dis. 2016; 11:1485–94. https://doi.org/10.2147/COPD.S105948 PMID: 27418822
22. Razzuoli R, Zanotti C, Amadori M. Chapter 8. Modulation of the Interferon Response by Environmental Noninfectious Stressors. In: Amadori M, editor. The Innate Immune Response to Noninfectious Stressors: Human and Animal Models. 1st Edition ed. San Diego: Elsevier Science Publishing Co Inc; 2016. p. 192–208.
23. Sonnenfeld G, Hudgens RW. Effect of sidestream and mainstream smoke exposure on in vitro interferon-alpha/beta production by L-929 cells. Cancer Res. 1986; 46(6):2779–83. PMID: 3698007.
24. Singanayagam A, Joshi PV, Mallia P, Johnston SL. Viruses exacerbating chronic pulmonary disease: the role of immune modulation. BMC Medicine. 2012; 10(1):27. https://doi.org/10.1186/1741-7015-10-27 PMID: 22420941
25. Simpson JL, Carroll M, Yang IA, Reynolds PN, Hodge S, James AL, et al. Reduced Antiviral Interferon Production in Poorly Controlled Asthma Is Associated With Neutrophilic Inflammation and High-Dose Inhaled Corticosteroids. Chest. 2016; 149(3):704–13. https://doi.org/10.1016/j.chest.2015.12.018 PMID: 26836898.
26. Thomas BJ, Porritt RA, Hertzog PJ, Bardin PG, Tate MD. Glucocorticosteroids enhance replication of respiratory viruses: effect of adjuvant interferon. Sci Rep. 2014; 4(7176):7176. https://doi.org/10.1038/srep07176 PMID: 25417801.
27. Divangahi M, King IL, Pernet E. Alveolar macrophages and type I IFN in airway homeostasis and immunity. Trends Immunol. 2015; 36(5):307–14. https://doi.org/10.1016/j.it.2015.03.005 PMID: 25843635.
28. Retamales I, Elliott WM, Meshi B, Coxson HO, Pare PD, Sciurba FC, et al. Amplification of inflammation in emphysema and its association with latent adenoviral infection. Am J Respir Crit Care Med. 2001; 164(3):469–73. https://doi.org/10.1164/ajrccm.164.3.2007149 PMID: 11500352
29. Barnes PJ. Cellular and molecular mechanisms of asthma and COPD. Clin Sci (Lond). 2017; 131(13):1541–58. https://doi.org/10.1042/CS20160487 PMID: 28659395.
30. Dewhurst JA, Lea S, Hardaker E, Dungwa JV, Ravi AK, Singh D. Characterisation of lung macrophage subpopulations in COPD patients and controls. Sci Rep. 2017; 7(1):7143. https://doi.org/10.1038/s41598-017-07101-2 PMID: 28769058.
31. Hogg JC. Pathophysiology of airflow limitation in chronic obstructive pulmonary disease. Lancet. 2004; 364(9435):709–21. https://doi.org/10.1016/S0140-6736(04)16900-6 PMID: 15325838
32. Makris S, Paulsen M, Johansson C. Type I Interferons as Regulators of Lung Inflammation. Front Immunol. 2017; 8(259):259. https://doi.org/10.3389/fimmu.2017.00259 PMID: 28344581.
33. Honda K, Yanai H, Takaoka A, Taniguchi T. Regulation of the type I IFN induction: a current view. Int Immunol. 2005; 17(11):1367–78. https://doi.org/10.1093/intimm/dxh318 PMID: 16214811
34. Perrotti E, Marsili G, Sgarbanti M, Remoli AL, Fragale A, Acchioni C, et al. IRF-7: an antiviral factor and beyond. Future Virol. 2013; 8(10):1007–20.
35. Fitzgerald-Bocarsly P, Dai J, Singh S. Plasmacytoid dendritic cells and type I IFN: 50 years of convergent history. Cytokine Growth Factor Rev. 2008; 19(1):3–19. https://doi.org/10.1016/j.cytogfr.2007.10.006 PMID: 18248767.
36. Schneider WM, Chevillotte MD, Rice CM. Interferon-Stimulated Genes: A Complex Web of Host Defenses. Annual review of immunology. 2014; 32:513–45. https://doi.org/10.1146/annurev-immunol-032713-120231 PMID: 24555472
37. Barral PM, Sarkar D, Su Z-z, Barber GN, DeSalle R, Racaniello VR, et al. Functions of the cytoplasmic RNA sensors RIG-I and MDA-5: Key regulators of innate immunity. Pharmacology & therapeutics. 2009; 124(2):219–34.

38. Baines KJ, Hsu AC, Tooze M, Gunawardhana LP, Gibson PG, Wark PA. Novel immune genes associated with excessive inflammatory and antiviral responses to rhinovirus in COPD. Respir Res. 2013; 14(15):15. https://doi.org/10.1186/1465-9921-14-15 PMID: 23384071.

39. Spann KM, Loh Z, Lynch JP, Ullah A, Zhang V, Baturcam E, et al. IRF-3, IRF-7, and IPS-1 promote host defense against acute human metapneumovirus infection in neonatal mice. Am J Pathol. 2014; 184(6):1795–806. https://doi.org/10.1016/j.ajpath.2014.02.026 PMID: 24726644.
